# Rabies vaccination adherence and associated factors among rabies-exposed patients in Shenzhen, China: a hospital-based cross-sectional study

**DOI:** 10.1017/S0950268824000049

**Published:** 2024-01-10

**Authors:** Ruiqi Lu, Jinsheng Lin, Yang Zhou, Qian Chen, Zaiying Fan, Shuning Wu, Pei Qin, Liping Li

**Affiliations:** 1 School of Public Health, Shantou University, Shantou, China; 2 Injury Prevention Research Center, Shantou University Medical College, Shantou, China; 3 Emergency Department, Shenzhen Qianhai Shekou Free Trade Zone Hospital, Shenzhen, China; 4 Department of Thyroid and Breast Surgery, Shenzhen Qianhai Shekou Free Trade Zone Hospital, Shenzhen, China; 5 Clinical Center for Public Health, Shenzhen Qianhai Shekou Free Trade Zone Hospital, Shenzhen, China

**Keywords:** adherence, influencing factors, post-exposure vaccination, rabies, urban area

## Abstract

Adherence to post-exposure prophylaxis and post-exposure vaccination (PEV) is an important measure to prevent rabies. The purpose of this study was to explore the adherence to the vaccination protocol and its influencing factors among rabies-exposed patients in Shenzhen, China. A cross-sectional survey was conducted in a tertiary hospital in Shenzhen, China, to obtain epidemiological characteristics of patients; knowledge, attitude, and practice scores of rabies prevention; and medical records. A total of 326 patients requiring full rabies PEV were included in this study, and only 62% (202) completed the full course of vaccination according to the norms of the vaccination guidelines. After multifactor logistic regression, the factors influencing adherence to vaccination were as follows: age 31 to 40 years, time spent to reach the nearest rabies prevention clinic was >60 min, the time of injury was at night to early morning, the place of injury was a school/laboratory, the animal causing injury was a cat, the health status of the animal causing injury could not be determined, and patients with higher practice scores (all *p*<0.05). Understanding the factors influencing rabies vaccination adherence among rabies-exposed patients in urban areas of China and promote changes in patients’ practice toward rabies prevention is essential for rabies elimination by 2030.

## Introduction

Rabies is the foremost lethal infectious zoonotic disease, with a 100% mortality rate in humans [1], and although the global disease burden of rabies has declined over the past 30 years, the number of global deaths from rabies in 2019 was still as high as 13,743 [2], especially in some developing countries and regions in Africa and Asia, accounting for 95% of all rabies cases, and the threat of rabies remains [3]. The World Health Organization, the World Organization for Animal Health, the Food and Agriculture Organization of the United Nations, and the Global Alliance for Rabies Control launched a global call in 2015 to eliminate human deaths due to rabies by 2030 [4, 5]. In China, dog-mediated rabies accounts for 95% of rabies cases nationwide, with the rest being mediated by cats and wildlife [6]. Although studies have demonstrated that mass vaccination of dogs against rabies is the most cost-effective option for rabies eradication [7], the current level of canine rabies vaccination in China is still low [8], especially in the less economically developed rural areas of China, and falls far short of the 70% mark [9, 10], so timely post-exposure prophylaxis (PEP) and post-exposure vaccination (PEV) for rabies-exposed patients remain important tools for rabies prevention and have important public health implications.

Currently, the two main rabies vaccination protocols in the world are the Essen five-dose method and the ‘2-1-1’ Zagreb four-dose method [11]; however, studies have shown that a significant proportion of rabies-exposed patients seeking PEP and receiving rabies PEV in a standardized manner are not highly compliant and have problems delaying or stopping vaccination on their own [12]. Failure to receive complete and standardized PEP or to follow recommended treatment protocol guidelines has also been associated with rabies immunization failure and mortality [13, 14]. Previous studies have mostly explored the adherence of rabies-exposed patients seeking PEP and rabies vaccination in areas where medical resources are scarce or in rural areas [15, 16]. However, studies have shown that residents of urban areas in China have poor knowledge of rabies and that compliance with PEVs among rabies-exposed patients in urban areas is suboptimal [17, 18]. This study combined the characteristics of animal injury occurrence and the level of rabies prevention knowledge, attitude, and practice (KAP) in rabies-exposed patients, aiming to illustrate the factors influencing adherence to PEV by rabies-exposed patients throughout the standardized process in Shenzhen and to provide data support and theoretical basis for improving rabies vaccination adherence among urban residents and standardizing the PEP and disposal process of rabies.

## Materials and methods

### Study setting, design, participants, and sampling

This study was conducted in the Nanshan District of Shenzhen City, China, which is one of the most developed cities in China and is representative of China’s emerging megacities with a highly mobile urban population. According to the Shenzhen Center for Disease Control and Prevention report, more than 100,000 people are bitten by dogs/cats in Shenzhen every year.

The study was conducted in Nanshan District, Shenzhen. Data were collected from a tertiary hospital, which is one of the four rabies PEP and rabies vaccination point hospitals in Nanshan District, Shenzhen, covering a population of nearly 800,000 people in a 65 square kilometre area, with a specialized outpatient clinic for the management of animal bites, which made it suitable for conducting the survey.

Considering the cross-sectional nature of the study and the fact that most of the rabies exposures in the urban population are caused by dogs/cats, we investigated the first-visit patients with injuries caused by dogs/cats and diagnosed by a specialized surgeon as having a rabies class II or III exposure (in countries and regions where rabies has not been eliminated, a rabies class II or III exposure means that the patient is at risk of rabies) who needed to undergo the full course of rabies PEV who were admitted to the hospital during the period of September 2022 to December 2022 and who were given electronic questionnaires by an investigator out of the principle of voluntarism and after their informed consent had been sought.

Prior to the start of the survey, hospital rabies vaccination records were randomly assessed to determine the sample size. Based on the results of the review, approximately 33% of patients failed to comply with the rabies PEP guidelines to complete the full course of PEV. Based on the cross-sectional study sample size calculation methodology, the estimated sample size was 340 at 80% power of the study, alpha = 0.05.

### Survey process

Before the formal survey, the researcher introduced the purpose of the study to the patients; the survey was conducted on a voluntary basis, and the questionnaires were filled out independently under the guidance of the surveyors or by their guardians if the patients were too young to complete the questionnaires. The questionnaires were collected by the ‘Wenjuanxing’ platform, which is the largest online questionnaire platform in China, with the advantages of easy sample collection and no access to the database by non-relevant personnel, and were completed by the patients using their smartphones and collected in the back-end database. The surveyors are all surgeons from the hospital’s emergency department and have good professionalism. During the survey period, the background data will be regularly reviewed by specialized personnel to ensure the validity of the survey information.

### Measure

#### Definition of adherence

Since the hospital’s rabies vaccination protocol was standardized to the ‘2-1-1’ Zagreb four-dose method, 2, 1, and 1 doses of rabies vaccine were administered on days 0, 7, and 21 after injury. For the purposes of this study, adherence was defined as the exact date of vaccination according to the vaccination protocol and non-adherence was considered if there was a delay or discontinuation of vaccination throughout the vaccination protocol.

#### Questionnaire design

The questionnaire was designed with good acceptability after referring to similar studies, taking into account the actual situation of the survey and consulting experts. The questionnaire consisted of four parts: the first part consisted of basic demographic information, including gender, age, education, income, history of dog and cat injuries, history of rabies vaccination, time spent to reach the nearest rabies prevention clinic (RPC), and payment method. The second part is the injury situation, including the injury site, exposure level, injury location, injury time, injury type, and injury cause. The third part is the situation of the injurious animal, including animal species, animal gender, animal source, animal vaccine history, and animal health status. The fourth part of the study was a rabies prevention KAP questionnaire, using a rabies KAP questionnaire from a study in central China [19], in which the rabies knowledge section consisted of 7 questions, including 1 multiple-choice question, with scores ranging from 0 to 9; the attitude section was a 5-point Likert scale with 9 questions and a score range of 9–45; and the practice section was a 4-point Likert scale with 6 questions and a score range of 6–24.

### Statistical processing

Data were organized using Excel and statistically analysed using SPSS 26.0. Descriptive analysis of basic characteristics was performed, and categorical variables were expressed using frequencies and composition ratios. Influence factor analysis was performed using variables screened based on the results of one-factor binary logistic regression analysis, and multifactor binary logistic regression was performed for variables in which the one-factor logistic regression was statistically significant; the continuous variables in the model were standardized. The two-sided test level was *α* = 0.05.

### Ethics statement

The study was approved by the Ethics Committee of Shenzhen Qianhai Shekou Free Trade Zone Hospital.

## Results

A total of 352 patients participated in the survey, with a final inclusion of 326 valid questionnaires and a valid response rate of 92.6%. By checking the patients’ medical records, a total of 202 respondents were finally confirmed to have received rabies PEV throughout the survey in a standardized manner, with an adherence rate of 62%.

### Socio-demographic characteristics of the survey respondents

The average age of the respondents was 28.52 years (SD = 13.562); more than half (55.5%) of the respondents were under 30 years old, 189 (58.0%) were female, 304 (93.3%) were permanent residents of Shenzhen, 208 (63.8%) were educated to college level or above, 190 (58.3%) had a per capita monthly household income of RMB 10,000 or above, the payment method was medical insurance for 162 (49.7%) patients, 149 (45.7%) patients were injured by dogs/cats for the first time, 177 (54.3%) had not received rabies vaccination, 279 (85.6%) were seen within 24 h, and 183 (56.1%) went to the nearest RPC in <30 min. Table 1 shows the sociodemographic characteristics of respondents.

**Table 1.** Socio-demographic characteristics of respondents (*n* = 326)

Characteristic	*n*	Percentage
Age group		
0–10	43	13.2
11–20	45	13.8
21–30	93	28.5
31–40	82	25.2
41–50	44	13.5
51–60	15	4.6
61–70	4	1.2
Sex		
Male	137	42.0
Female	189	58.0
Shenzhen permanent residents		
Yes	304	93.3
No	22	6.7
Education level		
Primary school and below	37	11.3
Junior high school	28	8.6
High school/junior college/technical school	53	16.3
College	67	20.5
Undergraduate	116	35.6
Graduate student and above	25	7.7
Monthly per capital household income		
Under 2,000 RMB	9	2.8
2,000–5,000 RMB	37	11.3
5,000–10,000 RMB	90	27.6
10,000 RMB or more	190	58.3
Payment method		
Medical insurance	162	49.7
Self-financed	156	47.9
Other	8	2.4
History of dog/cat injuries		
1st time	149	45.7
2 times	90	27.6
3 times and above	52	16.0
Do not remember	35	10.7
Rabies vaccination history		
Yes	149	45.7
No	177	54.3
Is it more than 24 h before the visit?		
Yes	47	14.4
No	279	85.6
Time spent to RPC		
<30 min	183	56.1
30 ~ 60 min	90	27.6
>60 min	53	16.3

RPC, rabies prevention clinic.

### Injuries of the surveyed patients

Among the 326 patients, 278 (85.3%) were exposed to rabies grade II, 222 (68.1%) were injured in the upper limbs, 207 (63.5%) were injured by scratching, 170 (52.1%) were injured in their own homes, and 161 (49.4%) were injured while playing or teasing with animals. Table 2 shows the details of the injuries.

**Table 2.** Injuries of the respondents (*n* = 326)

Characteristic	*n*	Percentage
Rabies exposure level		
II	278	85.3
III	48	14.7
Injury site		
Head, face, and neck	8	2.4
Trunk	11	3.4
Upper limbs	222	68.1
Lower limbs	84	25.8
Other	1	0.3
Injury type		
Bite injuries	86	26.4
Injure by scratching	207	63.5
Bites and scratches	21	6.4
Mucous membranes and wounds are licked	12	3.7
Injury time		
Morning	89	27.3
Afternoon	149	45.7
Night to early morning	88	27.0
Injury location		
Own home	170	52.1
Others’ homes	44	13.5
Public places	84	25.8
School/laboratory	28	8.6
Cause of injury		
Hurt by not being provoked	86	26.4
Injured while playing or teasing with animal	161	49.4
Attacked by rabid cats/dogs	8	2.5
Attacked while feeding food	34	10.4
Deliberate provocation leading to an attack	6	1.8
Unintentional injuries lead to their counterattack	31	9.5

### Injurious animals

Among the 326 patients, 242 (74.2%) were injured by cats and the rest by dogs; the animals were not vaccinated against rabies or the vaccine history was not clear for 109 (33.4%) patients; the animal health status was healthy for 199 (61.0%); and the source of injurious animals was self-owned for the largest proportion, 153 (46.9%). Table 3 shows the details of the injurious animals.

**Table 3.** Injurious animals (*n* = 326)

Characteristic	*n*	Percentage
Animal species		
Dog	84	25.8
Cat	242	74.2
Animal sex		
Male	124	38.0
Female	84	25.8
Unknown	118	36.2
Animal rabies vaccine history		
Yes	108	33.2
No	109	33.4
Unknown	109	33.4
Animal health status		
Healthy	199	61.0
Uncertain health status	127	39.0
Animal source		
Owned animals	153	46.9
Animals owned by others	89	27.3
Stray/wandering animals	71	21.8
Animals of unknown origin	13	4.0

### KAP of rabies prevention among survey respondents

The mean knowledge score of the 326 patients was 6.75 (SD = 1.786), the mean attitude score was 38.56 (SD = 6.517), the mean practice score was 17.89 (SD = 2.652), and the mean total score was 63.23 (SD = 8.698). Table 4 contains details on rabies prevention KAP score.

**Table 4.** Rabies prevention KAP score

	Mean	Median	95% CI
Knowledge^a^	6.75	7	1.786
Attitude^b^	38.56	41	6.517
Practice^c^	17.89	18	2.652
KAP total score^d^	63.23	65	8.698

a Knowledge section consists of 7 questions, with a total of 9 points and a score range of 0–9 points.

b Attitude part of a total of 9 questions, with a total of 45 points and a score range of 9–45.

c Practice part of a total of 6 questions, with a total of 24 points and a score range of 6–24.

d KAP total score of 78.

### Analysis of factors influencing vaccination adherence

Table 5 lists the factors influencing rabies vaccination adherence in patients with injuries caused by dogs and cats. Variables that were statistically significant in the one-way logistic regression analysis were included in the multifactor logistic regression analysis. After controlling for confounding factors, the results of the multifactorial logistic regression model indicated that non-adherence was more likely to occur in patients aged 31 to 40 years (OR = 3.024, 95% CI: 1.283–7.478) and in patients who spent >60 min to reach the nearest RPC (OR = 2.291, 95% CI: 1.109–4.803), while adherence was higher in patients who were injured at night to early morning (OR = 0.445, 95% CI: 0.219–0.888) and at places such as a school/laboratory (OR = 0.279, 95% CI: 0.073–0.926), whose injurious animal was a cat (OR = 0.505, 95%CI:0.273–0.925), and whose health status of the injurious animal could not be determined (OR = 0.355, 95% CI: 0.165–0.751). Adherence was also higher in patients with higher rabies prevention ‘practice’ scores (OR = 0.743, 95% CI: 0.570–0.962). Table 5 demonstrates the detailed results of the unifactorial and multifactorial analysis.

**Table 5.** Logistic regression analysis of factors influencing rabies vaccination adherence

	Univariable					Multivariable				
Characteristic	*N*	Event *N*	OR	95% CI	*P* value	*N*	Event *N*	OR	95% CI	*P* value
Gender										
Male	137	49	Ref.	–		137	49	Ref.	–	
Female	189	75	1.182	0.751, 1.868	0.472	189	75	1.309	0.785, 2.198	0.305
Age group										
0–10	43	12	Ref.	–		43	12	Ref.	–	
11–20	45	18	1.722	0.710, 4.290	0.233	45	18	2.366	0.872, 6.620	0.094
21–30	93	35	1.559	0.721, 3.519	0.269	93	35	2.081	0.882, 5.121	0.101
31–40	82	39	2.343*	1.078, 5.334	**0.036**	82	39	3.024*	1.283, 7.478	**0.013**
41–50	44	14	1.206	0.480, 3.061	0.690	44	14	1.064	0.381, 3.005	0.906
51–60	15	4	0.939	0.226, 3.380	0.926	15	4	0.746	0.161, 3.036	0.691
61–70	4	2	2.583	0.284, 23.612	0.369	4	2	4.398	0.382, 54.165	0.223
Time spent to reach RPC										
<30 min	183	62	Ref.	–		183	62	Ref.	–	
30–60 min	90	34	1.185	0.698, 1.998	0.526	90	34	1.190	0.664, 2.123	0.556
>60 min	53	28	2.186*	1.177, 4.086	**0.013**	53	28	2.291*	1.109, 4.803	**0.026**
Injury time										
Morning	89	38	Ref.	–		89	38	Ref.	–	
Afternoon	149	61	0.930	0.547, 1.588	0.790	149	61	0.848	0.466, 1.543	0.589
Night to early morning	88	25	0.533*	0.283, 0.990	**0.048**	88	25	0.445*	0.219, 0.888	**0.023**
Injury location										
Own home	170	75	Ref.	–		170	75	Ref.	–	
Other’s homes	44	19	0.963	0.488, 1.874	0.911	44	19	1.232	0.461, 3.319	0.677
Public places	84	25	0.537*	0.304, 0.929	**0.029**	84	25	0.951	0.397, 2.320	0.910
School/laboratory	28	5	0.275*	0.089, 0.705	**0.013**	28	5	0.279*	0.073, 0.926	**0.046**
Animal species										
Dog	84	42	Ref.	–		84	42	Ref.	–	
Cat	242	82	0.513**	0.309, 0.848	**0.009**	242	82	0.505*	0.273, 0.925	**0.028**
Animal health status										
Healthy	199	91	Ref.	–		199	91	Ref.	–	
Uncertain health status	127	33	0.417***	0.254, 0.672	**<0.001**	127	33	0.355**	0.165, 0.751	**0.007**
Animal source										
Owned animals	153	71	Ref.	–		153	71	Ref.	–	
Animals owned by others	89	31	0.617	0.357, 1.054	0.080	89	31	1.029	0.397, 2.636	0.953
Stray/wandering animals	71	19	0.422**	0.224, 0.770	**0.006**	71	19	1.336	0.464, 3.857	0.590
Animals of unknown origin	13	3	0.346	0.076, 1.184	0.118	13	3	1.134	0.188, 5.780	0.883
Score for ‘practice’^a^	326	124	0.729**	0.576, 0.916	**0.007**	326	124	0.743*	0.570, 0.962	**0.025**

a Score for ‘practice’: After standardization, the OR for each 1-unit increase in the standard deviation of the ‘practice’ score.

*P < 0.05, **P < 0.01; ***P < 0.001. Bold: statistically significant.

## Discussion

Rabies has a fatality rate of 100% but can be completely prevented if appropriate and effective PEP is administered [20]. However, many rabies-exposed patients fail to complete PEP and PEV on time for various reasons and often delay or stop vaccination on their own. This study was conducted in Shenzhen, one of the most economically developed cities in China, to explore the factors influencing rabies vaccination adherence among urban residents after rabies exposure with relatively easy access to healthcare resources, taking into account the characteristics of the occurrence of animal injuries.

Some studies have shown that the Zagreb method has a higher adherence rate compared to the Essen method due to its relatively easy vaccination procedure [21, 22], and the Zagreb method was adopted for all of this study, but its adherence rate only reached 62%. Although rabies science promotion has been carried out in China for many years and rabies vaccine is more available in metropolitan areas like Shenzhen, the standardized rabies PEV rate is still not satisfactory, and the adherence rate may be even lower in rural and less developed areas of China. In contrast to the results of previous studies [23, 24], patients’ education level and income levels do not seem to affect their rabies PEV adherence. According to the results of this study, respondents aged 31 to 40 years were more likely to not follow the time specified in the rabies vaccination guidelines, possibly because this age group is a prime workforce and often forgets the time in the vaccination protocol guidelines due to objective reasons such as busy work schedules [25] or is unable to reach the point hospital due to work [26], thus experiencing PEV delay or discontinuation. The time required to reach the nearest rabies clinic is also an important factor affecting adherence, and some respondents may not be able to receive rabies vaccination in a timely manner because they live too far away, have limited access to transportation, or do not know the exact location of the RPC near their place of residence, similar to the results of some studies that relate to the accessibility of medical services for rabies vaccination [27]. Adherence was higher in patients who were injured at night to early morning and at places such as schools/laboratories, suggesting that the time and place of injury may influence rabies vaccination adherence in patients with dog and cat injuries, but the mechanism of influence needs to be studied.

In addition, our study found that the species of injurious animal also influenced rabies vaccination adherence; in this study, the number of cases of patients injured by cats was much larger than those injured by dogs, but the adherence was higher in patients whose injurious animal was a cat, which is a matter of concern given the objective fact that dogs are the main vector of human rabies transmission in China. Adherence to rabies PEV was also higher when patients could not ensure that the injurious animal was in a healthy state, similar to the results of a study in Vietnam [28], suggesting a higher awareness of rabies prophylaxis after rabies exposure when patients were injured by an animal with an unclear health status. It is worth noting that in this study, although the level of ‘attitude’ toward rabies prevention was relatively high, the level of ‘practice’ scores was low and affected rabies PEV adherence. The higher the score, the higher the patient’s adherence to complete PEV, and it is necessary to promote behavioural changes in rabies prevention [29, 30], such as getting along properly with animals, understanding the severity of rabies exposure, and receiving PEV in a standardized manner.

Rabies prevention and control requires the collaboration of stakeholders from all aspects of society, including multisectoral cooperation between government departments and civil society organizations [31]. According to the concept of One Health, in order to reach the WHO goal of rabies elimination by 2030 [32, 33], a sound mechanism needs to be constructed among communities, hospitals, and animal regulatory authorities to strengthen residents’ scientific knowledge of rabies and PEV in communities; hospitals should standardize PEP disposal of rabies and increase the accessibility of rabies PEV for patients who need to receive it throughout the process to improve their adherence; and animal regulatory authorities need to improve animal registration and vaccination to ensure rabies vaccination of domestic animals, especially dogs and cats, to achieve the purpose of controlling rabies transmission by increasing the coverage of rabies vaccine in animals [34].

This study has some limitations. Firstly, data were collected through self-administered questionnaires, which may be subject to reporting bias. Secondly, although Shenzhen has reached 100% urbanization, the respondents of this study were solely from one hospital, and the sampling method was convenience-based potentially affecting sample representation.

PEP and rabies PEV are crucial elements of rabies prevention. It is recommended that health education on rabies and vaccination knowledge is integrated into the standardized approach for the management of animal injuries and rabies vaccination. This will encourage patients to adhere to the rabies vaccination protocol guidelines. By increasing awareness of PEV disposal clinic locations, improving accessibility of PEV services for patients, promoting changes in patient practice to prevent rabies, conducting further studies on the influence of animal injury characteristics on PEV adherence, and raising patients’ awareness of animal injury prevention – specifically the risk of dog and cat scratch bites, it may be possible to eliminate human rabies in China by 2030.

## Data Availability

All the data are available from the corresponding author upon reasonable request.
